# Health Risk Behaviour among Adolescents Living with HIV in Sub-Saharan Africa: A Systematic Review and Meta-Analysis

**DOI:** 10.1155/2018/7375831

**Published:** 2018-01-28

**Authors:** Derrick Ssewanyana, Patrick N. Mwangala, Anneloes van Baar, Charles R. Newton, Amina Abubakar

**Affiliations:** ^1^Centre for Geographic Medicine Research Coast, Kenya Medical Research Institute (KEMRI), P.O. Box 230, Kilifi, Kenya; ^2^Utrecht Centre for Child and Adolescent Studies, Utrecht University, P.O. Box 80140, 3508 TC Utrecht, Netherlands; ^3^Department of Psychiatry, Warneford Hospital, University of Oxford, Oxford OX3 7JX, UK; ^4^Department of Public Health, Pwani University, P.O. Box 195, Kilifi, Kenya

## Abstract

The burden of health risk behaviour (HRB) among adolescents living with HIV (ALWHIV) in sub-Saharan Africa (SSA) is currently unknown. A systematic search for publications on HRB among ALWHIV in SSA was conducted in PubMed, Embase, PsycINFO, and Applied Social Sciences Index and Abstracts databases. Results were summarized following PRISMA guidelines for systematic reviews and meta-analyses. Heterogeneity was assessed by the DerSimonian and Laird method and the pooled estimates were computed. Prevalence of current condom nonuse behaviour was at 59.8% (95% CI: 47.9–71.3%), risky sexual partnerships at 32.9% (95% CI: 15.4–53.2%), transactional sex at 20.1% (95% CI: 9.2–33.8%), and the experience of sexual violence at 21.4% (95% CI: 16.3–27.0%) among ALWHIV. From this meta-analysis, we did not find statistically significant differences in pooled estimates of HRB prevalence between ALWHIV and HIV uninfected adolescents. However, there was mixed evidence on the occurrence of alcohol and drug use behaviour. Overall, we found that research on HRB among ALWHIV tends to focus on behaviour specific to sexual risk. With such a high burden of HRB for the individuals as well as society, these findings highlight an unmet need for age-appropriate interventions to address the behavioural needs of these adolescents.

## 1. Introduction

Health risk behaviour (HRB) is a major concern in the prevention and management of HIV [1]. Such behaviour is often initiated or reinforced during adolescence [2]. The main forms of HRB include sexual behaviour contributing to unintended pregnancy and sexually transmitted diseases, alcohol, tobacco and drug use, unhealthy dietary habits, inadequate physical activity, and behaviour that contributes to unintentional injury or violence [3, 4]. Increased propensity for risk taking is a common phenomenon during adolescence [5]; adolescents living with HIV are vulnerable [6, 7]. They encounter various adverse impacts following their engagement in HRBs.

A number of studies conducted among sexually active adolescents living with HIV report that about a half have early sexual debut and unprotected sexual intercourse [6, 8–10]. Other studies have reported that adolescents living with HIV (ALWHIV) engage in various HRBs such as transactional sex, that is, sexual intercourse in exchange for material benefit or status [11, 12], alcohol abuse, and drug use [8, 13–15]. This is problematic for persons living with HIV, because such behaviour underlies suboptimal health outcomes such as poor adherence to antiretroviral treatment [16–18], HIV coinfection [19, 20], injury, and mortality [21]. Furthermore, this behaviour adversely impacts the socioeconomic welfare of affected families [22].

The occurrence of HRB among ALWHIV is of major public health significance in sub-Saharan Africa (SSA) where there was an estimated 1.2 million ALWHIV aged 15–19 years and 3.2 million HIV infected children below 15 years in 2014 [2]. The vulnerability to HRB and its consequences among the ALWHIV in SSA is exacerbated by the social environmental factors surrounding the HIV epidemic in this region. Among such factors are household poverty, orphanhood, gender inequality, stigma, cultural practices, and poor accessibility to social or health services [23–27]. Besides these factors, growing evidence suggests that underlying physiological conditions such as HIV associated neurodevelopmental deficits [28], anxiety, and depression [8, 10] increase susceptibility to risk taking among young people living with HIV.

In response to the enormous burden of HIV in SSA, some research and intervention programs have been conducted over the past few decades. Unfortunately such efforts have not addressed the needs of adolescents [38] although Africa is home to 19% of the global youth population [39]. Key among the research gaps is the scarcity of literature on HRB among adolescents living with HIV in SSA. Specifically, there is a dearth of knowledge regarding which forms of HRB have so far been assessed, characteristics of the ALWHIV (e.g., routes of HIV transmission), where such studies have been conducted in SSA and the general burden of HRB among the ALWHIV. The lack of such research is further compounded by combining the adolescent age group with other age categories [40] and the assessment of HRBs in isolation [41]. Upon this backdrop, this systematic review and meta-analysis aims at ascertaining the amount of research on HRB and documenting the general burden of HRB among adolescents living with HIV in SSA. The specific objectives are as follows:

- To identify and summarize characteristics of studies that quantify HRB among ALWHIV in SSA
- To summarize the major forms of HRB assessed among ALWHIV in SSA
- To compare the burden of HRB among ALWHIV and HIV uninfected adolescents among the eligible studies from SSA.

## 2. Methods

### 2.1. Search Strategy

Guidelines for preferred reporting items for systematic reviews and meta-analyses (http://www.prisma-statement.org/) were utilized [42]. Four databases (PubMed, Embase, PsycINFO, and Applied Social Sciences Index and Abstracts) were searched for publications from 1980 until 30th April 2016. We utilized the following key terms: (Adolescent*∗* OR Teen*∗* OR Youth*∗*) AND (Risk Taking OR Risk Behavior OR Risk Behaviour OR Life Style OR Health Behavior OR Health Behaviour) AND (HIV OR HIV/AIDS OR AIDS) AND (sub-Saharan Africa OR Africa). Additionally, a snowballing technique was applied by searching through reference lists of identified articles to access more articles which had not been identified from the database search.

We choose studies based upon the PICOS approach (participants, intervention, comparison, outcome, and study design) [42]. Studies were eligible if they (i) were empirical studies published in a peer-reviewed journal and conducted within SSA; (ii) involved ALWHIV whose age range, mean, or median age fell within 10–19 years; and (iii) quantified any form of HRB among the ALWHIV. We excluded studies that (i) were published in languages other than English and (ii) those that did not aggregate HRB by HIV status of the participants.

Two authors (DS and PNM) independently screened the titles, abstracts, and full articles for eligibility and reached consensus.

### 2.2. Data Extraction

We used one data extraction sheet to extract general study characteristics of the eligible studies. These characteristics included (i) author and year of publication; (ii) country where the study was done; (iii) year the study was done; (iv) study design; (v) population description; (vi) number of ALWHIV and HIV uninfected adolescents; (vii) route of HIV transmission; and (viii) form of HRB quantified.

Then, using two separate data extraction forms, we extracted the (i) author and year of publication; and (ii) data on each specific HRB. From each study HRB data for ALWHIV was extracted. However for the HIV uninfected adolescents this data was only extracted if it had been assessed as well among the ALWHIV. One form was used to extract data used in meta-analysis and the other for data that was to be narratively summarized. Data abstraction was conducted by two authors (DS and PNM) independently who then compared their results and reached consensus.

Our main outcome of interest for this systematic review and meta-analysis was the prevalence of specific HRBs among ALWHIV and HIV uninfected adolescents. For studies that were exclusively conducted among ALWHIV, we computed or extracted the reported percentages of those that engaged in a specific HRB. For those that mixed HIV infection groups and/or had additional age categories besides 10–19 years, we computed percentages of those that took part in a specified HRB for each HIV group within the 10–19 years age group. For those studies where it was impossible to compute these percentages, the occurrence of HRB was reported in its original effect measure, for example, odds ratio, median, or mean.

For each of the eligible studies, an assessment of the risk of bias across the studies was aided by the quality assessment tool for systematic reviews of observational studies (QATSO) [43]. The QATSO was designed for studies related to HIV prevalence or risky behaviour among men who have sex with men. It utilizes 5 parameters to obtain a total score that rates the overall quality of an observational study as either bad (0–33%), satisfactory (33–66%), or good (67–100%). These parameters include representativeness of sampling method used, objectivity of HIV measurement, report of participant response rate, control for confounding factors (in case of prediction or association studies), and privacy/sensitivity considerations. Each parameter is scored “1” if the condition was fulfilled and “0” if it was not.

### 2.3. Statistical Analysis

Data was synthesized both quantitatively and narratively. We assessed the variation in effect size attributable to heterogeneity using the *I*^2^ statistic of the DerSimonian and Laird method. Using random effects model, the pooled estimate was computed after Freeman-Tukey Double Arcsine transformation [44]. We compared the confidence intervals of the pooled estimates of the forms of HRB for the ALWHIV and HIV uninfected adolescents to determine if there were statistically significant differences. The statistical analyses were performed using STATA software (Stata Corporation, College Station, TX, 2005). We report the pooled estimates for four specific forms of HRB. These include the following:

- Current condom nonuse behaviour (including any reported episode of sexual intercourse without a condom for any duration that includes the current period, e.g., the last 3 months or last 6 months)
- Risky sexual partnerships (including reports of having 2 or more sexual partners currently or in the past 12 months or any form of multiple sexual partnerships)
- Sexual violence (including any reported episode (experienced or perpetrated) of forced sex, nonconsensual sex, or rape)
- Transactional sex (including any reported exchange of gifts or money for sex).

We narratively summarized the results that could not be quantitatively pooled (e.g., poor hygiene behaviour and alcohol and drug use behaviour) by describing the effect estimates such as percentages, odds ratios, mean with their standard deviations, and median with their interquartile ranges whatever reported in the study.

## 3. Results

**Figure 1:**
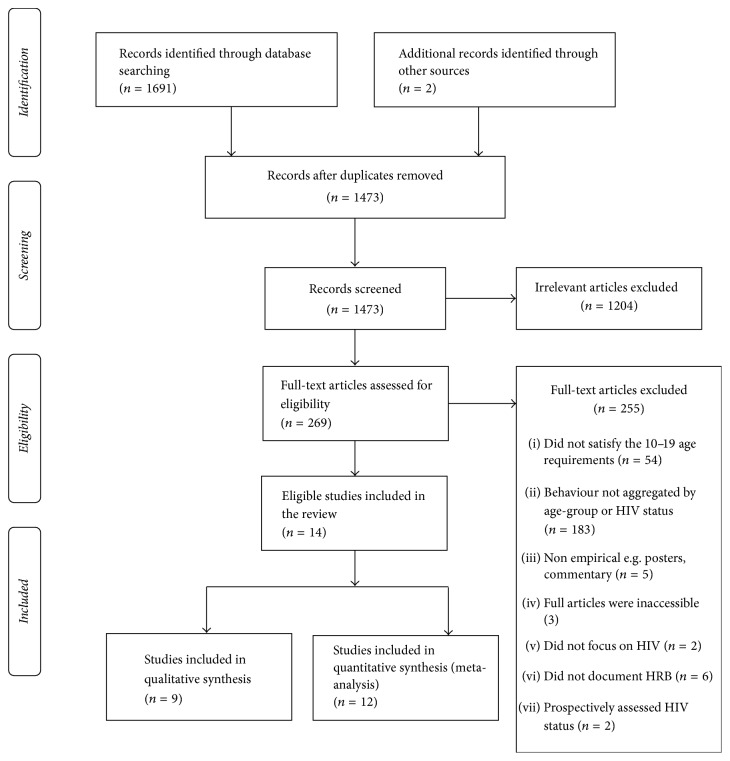
A flow diagram for the literature review.

We identified 1,691 published study citations from the 4 databases and an additional 2 articles [30, 37] through snowballing. Of these, 220 were duplicates. We therefore screened 1,473 abstracts for initial eligibility, out of which 269 articles were identified. Full articles were obtained for these citations, of which 14 satisfied the eligibility criteria (Figure 1).

The eligible studies were conducted between 1990 and 2012 among 6 sub-Saharan African countries of Nigeria, Rwanda, South Africa, Tanzania, Uganda, and Zimbabwe. The majority of the studies emanated from South Africa (*n* = 6) and Uganda (*n* = 4) of a total of 14. Most studies had a cross-sectional design, in addition two that utilized baseline data from a randomized control trial [13, 33] and another that used baseline data from a cohort study [37]. Samples of the ALWHIV per study ranged from 26 to 3,992 while those for HIV uninfected adolescents were from 296 to 6,600. Four studies [6, 14, 31, 35] had ALWHIV recruited from a clinical setting while the rest had their ALWHIV recruited from a general population setting through household surveys and community samples. Only three studies [6, 14, 31] described the route of HIV transmission among their participants. In these studies the majority (61–100%) had been perinatally infected (Table 1).

All the 14 eligible studies quantified sexual risk behaviour whereas alcohol use was quantified by 42.9%, sexual violence by 50.0%, and drug use by 21.4%. One study [30] assessed genital hygiene practices among male adolescents (Table 1). Among these 5 forms of HRB, sexual risky behaviour was the most variously assessed with specific examples like condom nonuse, transactional sex, sexual violence, dry sex practices (i.e., reducing vaginal lubrication to cause more friction during intercourse), early sexual debut, and multiple sexual partnerships. Details on specific HRB are summarized in Tables 2(a) and 2(b).

### 3.1. Sexual Risk Behaviour

Condom use behaviour was reported in 11 studies. We pooled results on current condom nonuse behaviour among ALWHIV from 9 studies and for HIV uninfected adolescents from 5 studies.

**Figure 2:**
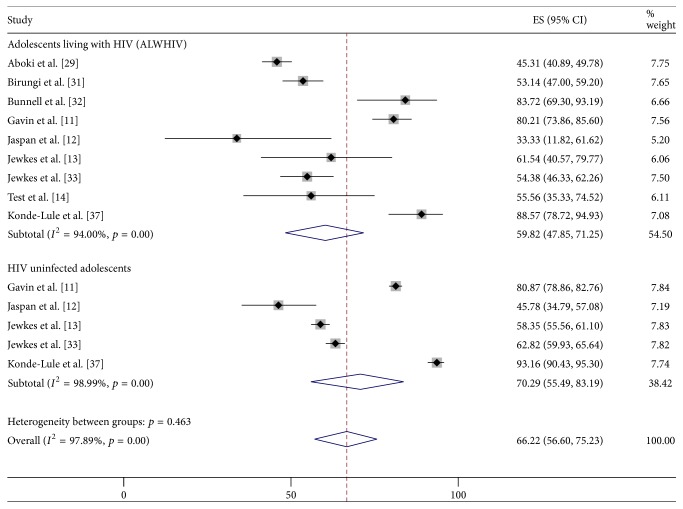
Prevalence of current condom nonuse behaviour among ALWHIV and HIV uninfected adolescents.

The pooled prevalence of condom nonuse behaviour among ALWHIV was estimated at 59.8% (95% CI: 47.9–71.3%) while among their HIV uninfected counterparts it was 70.3% (95% CI: 55.5–83.2%) (Figure 2). In contrast, findings from an additional study that was not part of the meta-analysis [30] reported a higher prevalence of condom nonuse at first sex among ALWHIV as compared to HIV uninfected adolescents (Table 2(b)).

**Figure 3:**
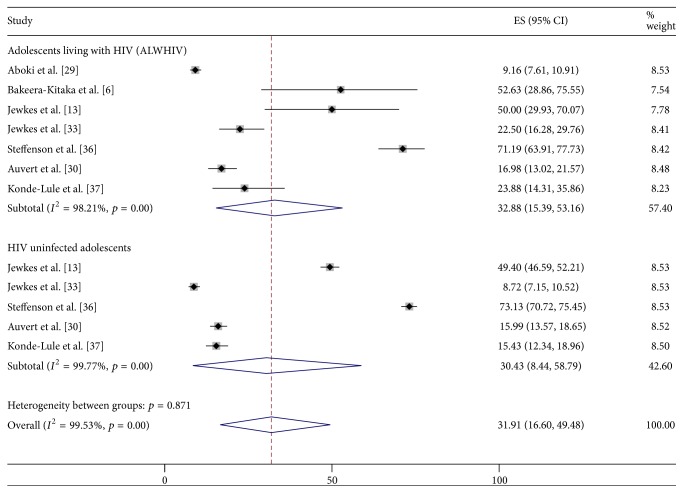
Prevalence of risky sexual partnerships among ALWHIV and HIV uninfected adolescents.

Additionally, the pooled prevalence of engagement in any form of risky sexual partnerships among ALWHIV was 32.9% (95% CI: 15.4–53.2%) whereas among HIV uninfected adolescents it was 30.4% (95% CI: 8.4–58.8%) (Figure 3).

Besides, there were four more studies capturing risky sexual partnerships that were not synthesized in our meta-analysis [11, 12, 34, 35] (Table 2(b)). One of them explored the association between HIV status and engagement in multiple sexual partnerships while comparing adolescents to young adults (aged 20–24 years) and found no statistically significant differences [35]. The second found no significant association between HIV status and having 6 or more sex partners in the past year among males who engaged in heterosexual anal sex [34]. The remaining 2 studies documented lifetime sexual partners among the adolescents of which one found that 4.7% of the ALWHIV compared to 1.4% of the HIV uninfected had more than 3 lifetime sexual partners [11] and the other reported a mean of 1.8 lifetime sexual partners among the ALWHIV compared to 0.7 among their HIV uninfected counterparts [12].

**Figure 4:**
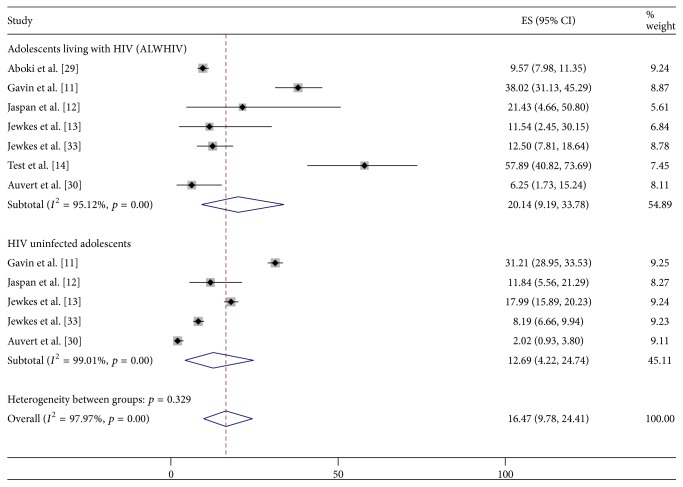
Prevalence of transactional sex among ALWHIV and HIV uninfected adolescents.

Transactional sex was prevalent among 20.1% (95% CI: 9.2–33.8%) of the ALWHIV and 12.7% (95% CI: 4.2–24.7%) of the HIV uninfected ones (Figure 4).

Another study [34] not included in this pooled estimate found no significant association between HIV status and purchasing sex among adolescents that reported heterosexual anal intercourse.

Early sexual debut among the ALWHIV was reported in 5 studies (Table 2(b)). Two of these studies reported that 25.5% [30] and 42.1% [6] of the ALWHIV initiated their first sex at the age of 15 years or less. Furthermore, a study from South Africa [13] and another from Rwanda [14] reported the median age at first sexual encounter as 14.7 (IQR: 12.9–16.2) and 17 (IQR: 15–18) years, respectively. A study among female ALWHIV reported a mean age of 16.4 (S.D: 0.1) years among the ALWHIV and 16.2 (S.D: 0.1) years among HIV uninfected adolescents at first sexual intercourse [11].

Two studies reported a 6.2% prevalence of dry sex practices (i.e., reducing vaginal lubrication to cause more friction during intercourse) among female ALWHIV. In both studies, the prevalence of dry sex practices was lower among the HIV uninfected adolescents [30, 33] (Table 2(b)). Another study [31] reported high prevalence of none contraceptive use at either first sex (63%) or during current or previous relationships (48%) among ALWHIV (Table 2(b)).

### 3.2. Alcohol and Drug Use

Six studies quantified alcohol and drug use behaviour (Table 2(b)). All of the 6 studies reported alcohol drinking behaviour of which 3 compared ALWHIV and uninfected adolescents. Among the 3 studies with results for both HIV groups [11, 13, 33] the ALWHIV recorded higher occurrence of alcohol drinking behaviour (Table 2(b)). Another study reported that 61% of ALWHIV receiving medication from a clinic had drunk alcohol within 6 hours prior to having sex [14]. In another study among males who engaged in heterosexual anal sex, HIV status was not significantly associated with having anal sex under the influence of alcohol [34].

Drug use behaviour was reported by 3 studies. One reported its occurrence among 53.8% of the male ALWHIV compared to 38.1% of their HIV uninfected male counterparts [13]. The same authors in another study [33] reported the occurrence of drug use among 5.0% of the female ALWHIV compared to 6.3% of the HIV uninfected ones. The third study reported drug use among males who had heterosexual anal sex and showed that there was not a significant association between HIV status and heterosexual anal sex under the influence of drugs [34].

### 3.3. Sexual Violence

**Figure 5:**
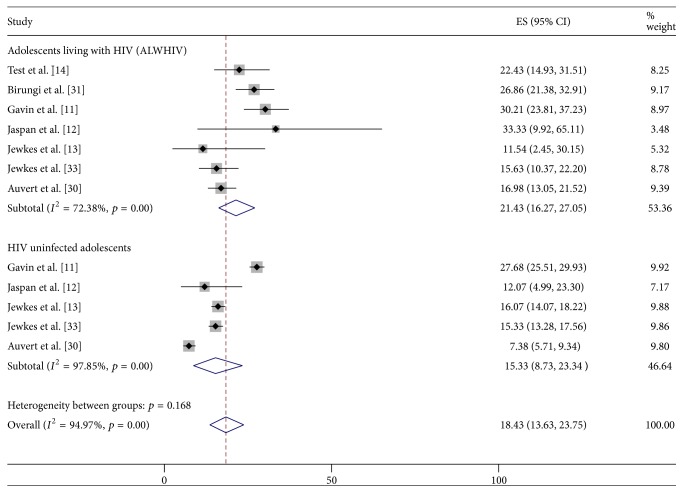
Prevalence of sexual violence behaviour among ALWHIV and HIV uninfected adolescents.

Seven studies captured reports of various forms of sexual violence such as forced sex, tricked sex, nonconsensual sex, and rape. Six of these studies [11, 12, 14, 30, 31, 33] specifically reported victims' experience of sexual violence while only one study [13] conducted among rural South African males reported perpetrators' experience of sexual violence. The pooled prevalence of any form of sexual violence (i.e., either as a victim or as perpetrator) was 21.4% (95% CI: 16.3–27.0%) among ALWHIV, while that among HIV uninfected adolescents was 15.3% (95% CI: 8.7–23.3%) (Figure 5).

*Poor hygiene behaviour* was documented in one study from a small mining town in South Africa which reported that 22.5% of the male ALWHIV compared to 11.4% of HIV uninfected males did not wash their genitals at least once a day [30].

Overall, the studies were of high quality with 10 of them rating as good and the remaining 4 [6, 13, 33, 34] as satisfactory. Only 2 of the studies utilized nonprobability sampling [6, 31], 6 did not report the participant response rate [6, 12, 13, 33–35], and 3 did not mention how privacy or sensitivity of HIV was considered in the study [13, 33, 34].

## 4. Discussion

This review indicates that research on HRB among adolescents living with HIV in SSA is still scanty. Moreover, within SSA, this research emanates from a few countries in eastern and southern Africa. The within region variation possibly represents disparities in HIV burden such that most of this research has so far focused on parts of SSA with higher HIV prevalence, for example, southern Africa. However, since SSA globally accounts for the largest population of ALWHIV [2], there is an urgent need for more research on HRB of this population.

Furthermore, even among the few existing studies, important details such as the route of transmission and the adolescents' awareness of their HIV status are scanty and yet these are potential determinants of behavioural decision making [45]. The participants are also mainly drawn from the general population or clinical setting. However, it is likely that adolescents from certain settings, for example, dwellers of fishing communities and busy transport corridors, would report a disproportionately higher burden of HRB since such settings are associated with high HIV sociobehavioural risk [46, 47].

Owing to the overlapping confidence intervals of effect estimates, our findings indicate that there is no statistically significant difference in the prevalence of documented forms of HRB across the ALWHIV and HIV uninfected adolescent groups in SSA. This stated that the prevalence of these HRB is high among both groups which stresses a major and so far unmet need for intervention among adolescents. The consequences of HRB in terms of psychosocial burden, injury, morbidity, and mortality are enormous [48]. Moreover, for ALWHIV, these may be exacerbated by their compromised health condition coupled with their increased need for optimizing care and treatment outcomes [17, 19, 20].

The high occurrence of unprotected sex at both current and first sexual intercourse among these adolescents is a serious concern. This is moreover compounded by concurrent sexual partnerships, transactional sex, and sexual related violence in the form of nonconsensual sex, intimate partner violence, and rape which are comparably high among both the ALWHIV and HIV uninfected adolescents. Similar to results from this review, some cross-sectional studies from the USA have documented a high prevalence of unprotected sex of 65% [10] and 62% [9] among adolescents living with HIV. Another systematic review of studies from SSA also indicates that transactional sex is a significant risk factor for HIV infection especially among young women [49]. Our findings on prevalence of sexual violence are within the ranges reported among adolescent girls from SSA [50]. This burden is similar for both ALWHIV and their uninfected counterparts but most importantly is that this is an unacceptably high burden for both groups. We suggest that high occurrence of risky sexual behaviour, sexual violence, and other forms of potentially high risk sexual practices such as transactional sex among ALWHIV may partly result from their vulnerable background that often is characterized by stigma, psychological vulnerability, family stressors, poverty, and orphanhood [23, 51]. Additionally, some underlying physiological pathways such as neurodevelopmental deficits, mental health, and HIV comorbidities possibly elucidate some behavioural trends.

Furthermore, our findings reveal that the use of alcohol and drugs is largely problematic especially among male adolescents in SSA. Similar to our findings, a number of studies from other regions have reported a similar problem of alcohol and drug use including among male adolescents living with HIV [10, 52]. The use of alcohol and drugs among people living with HIV is linked to numerous problems like poor adherence outcomes [16], psychiatric comorbidity [53], and HIV infection [54]. More so, drug and alcohol use may form a niche for impulsivity and aggravated risk taking such as intimate partner violence, rape, and unprotected sex, among others [55, 56].

Our results highlight much needed efforts of increasing research on HRB among ALWHIV in SSA, broadening the scope of HRBs currently being explored and including adolescents from most at-risk settings among such studies. Additionally, it is necessary to target ALWHIV with pragmatic interventions that address their specific needs so as to prevent or reduce their engagement in HRBs. These interventions also need to foster safe and healthy environments in which adolescents do not fall victim to HRBs and forms of sexual injustices such as sexual violence and transactional sex.

One of the limitations of our review is that HRB is self-reported among all the eligible studies and this may have involved some degree of social desirability bias. This form of bias generally arises when respondents answer questions in a way that favours their impression management [57]. However, assessment of HRB is predominantly conducted through self-reports. Additionally, our research focus was limited to studies conducted in SSA and thus generalizability of our results to the entire African and other geographical contexts should only be done with caution.

## 5. Conclusion

Research on HRB among adolescents living with HIV in SSA is still limited and currently focuses on a few forms of HRB especially behaviour specific to sexual risk. Nonetheless, the existing research from this region reveals an appalling burden, especially of sexual violence (where in most cases the adolescents are victims), sexual risk behaviour, and substance or drug use. While HRB is noted to compromise health outcomes, the studies do not report a number of factors such as route of HIV transmission and awareness of HIV status which could enhance our understanding of the context of HRB in this patient group. Furthermore, the assessment of HRB is not uniform pointing to the need for utilization of standardized assessment tools that would ensure better comparability of findings across studies. Nonetheless, the current review provides important insights into future research in the field of health risk behaviour and highlights the urgent need for age-appropriate interventions that will effectively address the behavioural and health needs of adolescents living with HIV in SSA. The ALWHIV themselves do not engage less in HRB than HIV uninfected adolescents. We suggest that further research is needed to explore in depth the forms of HRB and their predisposing and protective factors among ALWHIV and HIV uninfected adolescents within the SSA context. Such research may be crucial in guiding intervention planning for HRB and ensuring that the interventions are responsive to special needs and challenges faced by specific adolescent groups like ALWHIV, for example, stigma, depression, and orphanhood [16, 17, 23, 24].

## Figures and Tables

**Table 1:** Description of studies included in the systematic review and meta-analysis.

First author	Country	Year of study	Population description	ALWHIV	HIV uninfected adolescent participants	Route of HIV transmission	Form of HRB	Quality Assessment (QATSO score)
Aboki [29]	Nigeria	2007, 2012	Adolescent participants in a national survey	1,980 (in 2007) 3,992 (in 2012)	NA	NR	(i) Sexual risk behaviour	80% (Good)

Auvert [30]	South Africa	1999	Sexually active youths living in a small mining town	325	857	NR	(i) Alcohol use behaviour (ii) Sexual risk behaviour (iii) Sexual violence (iv) Hygiene practices	100% (Good)

Bakeera-Kitaka [6]	Uganda	2006	ALWHIV from a pediatrics clinic	75	NA	86% (perinatal) 2.6% (blood transfusion) 1.3% (Heterosexual) 9.3% (unknown)	(i) Sexual risk behaviour	50% (Satisfactory)

Birungi [31]	Uganda	2007	ALWHIV from various HIV/AIDS treatment programs	732	NA	Perinatal	(i) Sexual risk behaviour (ii) Sexual violence	75% (Good)

Bunnell [32]	Uganda	2004-2005	Adolescent participants from a household survey	43	NA	NR	(i) Sexual risk behaviour	100% (Good)

Gavin [11]	Zimbabwe	2001-2002	Adolescent females from a household survey	192	1,615	NR	(i) Sexual risk behaviour (ii) Alcohol use behaviour (iii) Sexual violence	100% (Good)

Jaspan [12]	South Africa	2004-2005	Adolescents participants from a house hold survey	35	296	NR	(i) Sexual risk behaviour (ii) Sexual violence	80% (Good)

Jewkes [13]	South Africa	2002-2003	Males from a rural setting	26	1,252	NR	(i) Sexual risk behaviour (ii) Alcohol use behaviour (iii) Drug use behaviour (iv) Sexual violence	60% (Satisfactory)

Jewkes [33]	South Africa	2002-2003	Females from a rural setting	160	1,135	NR	(i) Alcohol use behaviour (ii) Drug use behaviour (iii) Sexual risk behaviour (iv) Sexual violence	60% (Satisfactory)

Lane [34]	South Africa	2003	Males who engage in heterosexual anal sex (data from household survey)	NR	NR	NR	(i) Sexual risk behaviour (ii) Alcohol use behaviour (iii) Drug use behaviour	60% (Satisfactory)

Mhalu [35]	Tanzania	2010	ALWHIV receiving care	110	NA	NR	(i) Sexual risk behaviour	80% (Good)

Steffenson [36]	South Africa	2003	Adolescent participants from a household survey	1,092	6,600	NR	(i) Sexual risk behaviour	100% (Good)

Test [14]	Rwanda	2010	Adolescents receiving medication from an ART clinic	68	NA	61% (perinatal) 33% (Sexually) 5% (others)	(i) Sexual risk behaviour (ii) Alcohol use behaviour (iii) Sexual violence	80% (Good)

Konde-Lule [37]	Uganda	1990	Adolescents from a household survey	70	499	NR	(i) Sexual risk behaviour	100% (Good)

ALWHIV were adolescents (10–19 years) that were living with HIV in each of the eligible study; NR refers to “not reported”; NA refers to “not applicable.”

**Table:** (a) Description of health risk behaviour of ALWHIV and HIV uninfected adolescents among studies included in the meta-analysis

First author	Condom non use % (total respondents)	Transactional sex % (total respondents)	Risky sexual partnerships % (total respondents)	Sexual violence % (total respondents)
Aboki [29]	44.7 (501) of ALWHIV did not use a condom with a non-marital partner at last sex from data collected in 2012	9.5 (1,223) of ALWHIV engaged in transaction sex from data collected in 2012	9.2 (1,234) of ALWHIV had multiple non-marital partners from data collected in 2012	NA

Auvert [30]		6.3 (64) of male ALWHIV had ever given money in exchange for sex 1.9 (446) of HIV uninfected adolescent males had ever given money in exchange for sex	16.9 (318) of ALWHIV had 2 or more current sexual relationships 15.9 (838) of HIV uninfected adolescents had 2 or more current sexual relationships	16.9 (324) of ALWHIV ever had sex against his/her will 7.4 (854) of HIV uninfected adolescents ever had sex against his/her will

Bakeera-Kitaka [6]	NA	NA	52.8 (19) of ALWHIV had more than 2 sexual partners	NA

Birungi [31]	53 (271) of ALWHIV who ever had sex were currently not using condoms	NA	NA	27 (242) of ALWHIV ever had sex against his/her will

Bunnell [32]	83.7 (43) of ALWHIV did not always use a condom with all most recent partners in the last 12 months	NA	NA	NA

Gavin [11]	80.3 (192) of ALWHIV did not use a condom at last sex 80.9 (1,615) of HIV uninfected adolescents did not use a condom at last sex	37.9 (192) ALWHIV exchanged goods for sex with last partner 31.2 (1,615) of HIV uninfected adolescents exchanged goods for sex with last partner	NA	29.9 (192) of ALWHIV were ever forced to have sex 27.7 (1,615) of HIV uninfected adolescents were ever forced to have sex

Jaspan [12]	33.3 (15) of sexually active ALWHIV had never used a condom 45.8 (83) of HIV uninfected sexually active adolescents had never used a condom	21.4 (14) of ALWHIV ever received a gift in exchange for sex 12 (76) of HIV uninfected adolescents ever received a gift in exchange for sex	NA	33 (12) of female ALWHIV reported forced sex from their boyfriends 12 (58) of HIV uninfected females reported forced sex from their boyfriends

Jewkes [13]	61.5 (26) of ALWHIV did not correctly use a condom during last sex 58.4 (1,251) of HIV uninfected adolescents did not correctly use a condom during last sex	11.5 (26) of ALWHIV had transactional sex with a casual partner 18.0 (1,251) of HIV uninfected adolescents had transactional sex with a casual partner	50 (26) of ALWHIV had 3 or more consenting partners in the last year 49.4 (1,251) of HIV uninfected adolescents had transactional sex with a casual partner	11.5 (26) of male ALWHIV reported having raped a non-partner 16.1 (1,251) of male HIV uninfected adolescents males reported having raped a non-partner

Jewkes [33]	54.4 (160) of ALWHIV did not correctly use a condom during last sex 62.8 (1,135) of HIV uninfected adolescents did not correctly use a condom during last sex	12.7 (160) of ALWHIV had transactional sex with a casual partner 8.2 (1,135) of HIV uninfected adolescents had transactional sex with a casual partner	22.5 (160) of ALWHIV had 3 or more consenting partners in the last year 8.7 (1,135) of HIV uninfected adolescents had 3 or more consenting partners in the last year	15.6 (160) of female ALWHIV reported having been forced or tricked in to first sex 15.3 (1,135) of HIV uninfected adolescent females reported having been forced or tricked in to first sex

Steffenson [36]	NA	NA	71.2 (177) of ALWHIV had at least one concurrent sexual partnership in the past 12 months 73.1 (1,392) HIV uninfected adolescents had at least one concurrent sexual partnership in the past 12 months	NA

Test [14]	55.6 (27) of ALWHIV reported inconsistent condom use in past 6 months	57.9 (38) of ALWHIV had exchanged sex for money		22.4 (107) of ALWHIV had experienced forced sex

Konde-Lule [37]	88.6 (70) of ALWHIV report that they do not use condoms 93.2 (453) HIV uninfected adolescents report that they do not never use condoms	NA	23.9 (67) ALWHIV had 2 or more partners in the past year 15.4 (486) HIV uninfected adolescents had 2 or more partners in the past year	NA

NA refers to “not applicable”; total respondents refer to number of adolescents (10–19) that responded to a particular item assessing HRB.

**Table:** (b) Description of health risk behaviour of ALWHIV and HIV uninfected adolescents among studies included in the narrative review

First author	Condom non-use	Transactional sex	Risky sexual partnerships	Alcohol & Drug use	Early sex debut	Others
Auvert [30]	82.5 (325) of ALWHIV reported condom non-use at first sex 75.9 (854) HIV uninfected adolescents reported condom non-use at first sex	NA	NA	28.9% of 325 ALWHIV drank alcohol at least once a month	25.5% of 325 ALWHIV had first sex at 15 or less years	6.2% of 258 female ALWHIV engaged in dry sex practices 22.5% of 62 male ALWHIV did not wash their penis at least once a day 3.9% of 359 HIV uninfected female adolescents engaged in dry sex practices 11.4% of 491 HIV uninfected males did not wash their penis at least once a day

Bakeera-Kitaka [6]	NA	NA	NA	NA	42.1% of 19 ALWHIV initiated sex at below 15 years	NA

Birungi [31]						63% of 87 ALWHIV did not use a method to prevent infection or reinfection at first sex 48% of the ALWHIV did not use any form of contraception in current or previous relationship

Gavin [11]	NA	NA	4.7% of 192 ALWHIV reported more than 3 lifetime sexual partners 1.4% of 1,615 HIV uninfected adolescents reported more than 3 lifetime sexual partners	5.6% of 192 ALWHIV drank alcohol in past month 4.5% of 1,615 HIV uninfected adolescents drank alcohol in past month	Mean age at sex initiation was 16.4 years (SD: 0.14) among ALWHIV Mean age at sex initiation was 16.2 years (SD: 0.10) among HIV uninfected adolescents	NA

Jaspan [12]	NA	NA	Mean number of 1.8 life partners among the ALWHIV Mean number of 0.7 life partners among the HIV uninfected adolescents	NA	NA	NA

Jewkes [13]	NA	NA	NA	30.8% of 26 ALWHIV reported problem alcohol drinking 53.8% of 26 ALWHIV reported ever using drugs 25.8% of 1,251 HIV uninfected adolescents reported problem alcohol drinking 38.1% of 1,251 HIV uninfected reported ever using drugs	Median age sex initiation was 14.7 years (IQR: 12.9–16.2) among ALWHIV (i) Median age sex initiation was 14.7 years (IQR: 12.9–16.2) among HIV uninfected adolescents	NA

Jewkes [33]	NA	NA	NA	3.8% of 160 ALWHIV reported problem alcohol drinking 5% of 160 ALWHIV reported ever using drugs 3.5% of 1,135 HIV uninfected adolescents reported problem alcohol drinking 6.3% of 1135 HIV uninfected adolescents reported ever using drugs	NA	6.2% of 160 female ALWHIV engaged in dry sex practices 4.1% of 1,135 female HIV uninfected adolescents engaged in dry sex practices

Lane [34]	NA	aOR = 0.8^*∗*^ for HIV infection among male sex purchasers	aOR = 0.3^*∗*^ for HIV infection among males with 6 or more partners in past year	aOR = 1.2^*∗*^ for HIV infection among males that had sex under influence of alcohol aOR = 1.3^*∗*^ for HIV infection among males that had sex under influence of drugs	NA	NA

Mhalu [35]	aOR = 2.7^*∗∗*^ for having unprotected sex at last sexual contact among ALWHIV of 15–19 vs 20–24 years	NA	aOR = 1.2^*∗*^ for engaging in multiple sexual partnerships among ALWHIV of 15–19 vs 20–24 years	NA	NA	NA

Test [14]	NA	NA	NA	61% of 18 ALWHIV drank alcohol within 6 hours prior to sex	Median age sex initiation was 17 years (IQR: 15–18)	NA

NA refers to “not applicable”; aOR: adjusted odds ratio; ^*∗∗*^statistically significant; ^*∗*^not statistically significant; IQR: interquartile range SD: standard deviation.
